# Sex-different and growth hormone-regulated expression of microRNA in rat liver

**DOI:** 10.1186/1471-2199-10-13

**Published:** 2009-02-23

**Authors:** Louisa Cheung, Carolina Gustavsson, Gunnar Norstedt, Petra Tollet-Egnell

**Affiliations:** 1 Department of Molecular Medicine and Surgery, Karolinska Institutet, Centre for Molecular Medicine, L8:01, Karolinska University Hospital, 171 76 Stockholm, Sweden

## Abstract

**Background:**

MicroRNAs (miRNAs) are short non-coding RNAs playing an important role in post-transcriptional regulation of gene expression. We have previously shown that hepatic transcript profiles are different between males and females; that some of these differences are under the regulation of growth hormone (GH); and that mild starvation diminishes some of the differences. In this study, we tested if hepatic miRNAs are regulated in a similar manner.

**Results:**

Using microarrays, miRNA screening was performed to identify sex-dependent miRNAs in rat liver. Out of 324 unique probes on the array, 254 were expressed in the liver and eight (3% of 254) of those were found to be different between the sexes. Among the eight putative sex-different miRNAs, only one female-predominant miRNA (miR-29b) was confirmed using quantitative real-time PCR. Furthermore, 1 week of continuous GH-treatment in male rats reduced the levels of miR-451 and miR-29b, whereas mild starvation (12 hours) raised the levels of miR-451, miR-122a and miR-29b in both sexes. The biggest effects were obtained on miR-29b with GH-treatment.

**Conclusion:**

We conclude that hepatic miRNA levels depend on the hormonal and nutritional status of the animal and show that miR-29b is a female-predominant and GH-regulated miRNA in rat liver.

## Background

MicroRNAs (miRNAs) are short RNAs of 19–25 base pairs in length. In the latest release of miRBase (11.0, April 2008), a sequence database of miRNA hosted by the Sanger Institute [1], there are 678 identified and known miRNAs in human, 472 in mouse and 287 in rat. Most of the well-defined miRNAs are highly conserved across species, suggesting their importance in cellular regulation. These small RNAs are processed from precursor molecules that are either transcribed as ordinary genes (pri-miRNA) or generated as by-products from splicing. The maturation takes place in the cytoplasm when the miRNAs are assembled into miRNA-ribonuclear protein complexes, which in turn selectively bind to a target mRNA and modulate its translatability. At least five possible mechanisms for this miRNA-dependent regulation of translation have been suggested [2,3]. If the sequences of the miRNA and the target mRNA are a perfect match it will i) trigger endonucleic cleavage. If the sequences are an imperfect match the miRNA will instead ii) inhibit translation by promoting deadenylation of the poly(A) tail, or iii) promote degradation of the nascent peptide, or iv) repress the assembly of ribosomal complex for translation initiation, or v) reduce the speed of the elongation process. Independently of their mechanisms of action, they play a role in gene regulation by negatively affecting mRNA expression and translation.

Some miRNAs are ubiquitous, such as let-7b and miR-22 [4], while others are highly tissue specific. miR-1a is heart-specific and miR-124a is exclusively found in nervous tissues such as brain and spinal cord [4,5]. miR-122a is abundant in liver, where it contributes with 70% of the total pool of hepatic miRNA. Studies have shown that miR-122a is a regulator of hepatic lipid metabolism, since inhibition of this miRNA lowers the serum cholesterol levels in normal mice and mice with diet-induced obesity. At the cellular level, miR-122a increases hepatic fatty acid oxidation and decreases lipogenesis [6]. Although the molecular mechanisms behind these effects are still unclear, these findings have paved the way for a new concept within the field of metabolic research.

The liver is a major metabolic organ, handling the metabolism of exogenous and endogenous compounds. We and others have shown that male and female rat livers differ regarding gene expression and metabolism [7-10]. We have previously shown that genes involved in hepatic lipid metabolism are regulated by sex and hormones [11-13] as well as nutrient availability [11]. Based on microarray-generated hepatic transcript profiles, approximately 30% of the sex-differentiated hepatic gene products were shown to be under the control of growth hormone (GH) [11], and others have shown that the sex-different secretory pattern of GH in rats are mediating at least some of these effects [14-16]. Nothing is known about sex-differences in miRNA levels or if they vary depending on hormonal or nutritional status. In this study, we tested the possibility that miR-122a or other miRNAs from rat liver are expressed differently between males and females and if they are regulated by GH or starvation.

## Results

### Sex-differences in hepatic miRNA profiles

Initially, we screened for miRNAs with a sex-different expression using microarray analysis. We generated hepatic miRNA profiles from normal male and female rats. Out of 324 unique probes on the array, 254 (78% of 324) were expressed in the liver and eight (3% of 254) of those were found to be different between the sexes (Table 1). Among the sex-different miRNAs listed in the table, only miR-21, miR-29b and miR-122a have been shown to be expressed in liver before [4,6,17-22], implying that the other five might be "new" hepatic miRNAs. However, miR-451, miR-148a and miR-193 have recently been detected in liver by a high-throughput sequencing approach (Sarah Leigh-Brown, personal communication).

**Table 1:** Sex-differently expressed miRNAs in rat liver

	miRNA ratios comparing individual female and male livers (n = 4)						
miRNA	1	2	3	4	average	p-value	q-value
Male-predominant							
miR-451	0.64	0.86	0.42	0.51	**0.6**	< 0.001	6.824
miR-148a	0.70	0.82	0.67	0.67	**0.7**	< 0.001	6.824
miR-21	0.84	0.66	0.80	0.63	**0.7**	0.002	6.824
miR-526c	0.82	0.72	0.71	0.69	**0.7**	< 0.001	6.824
Female-predominant							
miR-205	2.70	2.40	1.84	0.97	**2.0**	0.043	6.824
miR-193a	1.40	1.53	1.82	1.48	**1.6**	< 0.001	6.824
miR-29b	1.52	1.58	1.53	1.42	**1.5**	< 0.001	6.824
miR-122a	1.04	1.38	1.99	1.52	**1.5**	0.050	8.620

The table was generated from a comparison of miRNA expression in livers from male and female rats, using microarrays (as described in the materials and methods section). miRNAs showing at least 1.5 fold sex-differences in expression levels, with a q-value (false discovery rate) less than 10%, were considered to be significantly sex-different. The data is presented as the ratio between female and male expression levels. A p-value is included comparing the overall ratio to a value of 1.0, using student's t-test.

**Figure 1:**
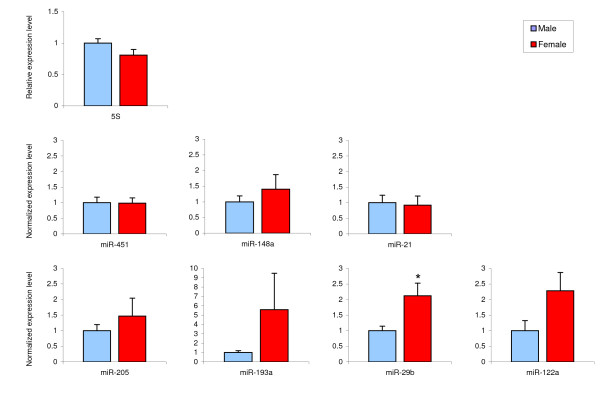
**Real-time PCR analysis of miRNA levels in male and female rat liver**. Male and female rat livers were used to extract miRNA-enriched RNA. cDNA was synthesized from 100 ng of this miRNA-enriched RNA using miRNA specific primers (miR-29b, miR-451, miR-122a) or random hexamers (5S rRNA), and real-time PCR analysis was used to quantify RNA levels. The results were related to corresponding levels in male livers and presented as ratios (mean ± S.E.). * indicate significant differences between males and females (p < 0.05), using student's t-test.

To validate our findings, miRNAs from Table 1 were validated using quantitative real-time PCR (qRT-PCR). miR-526c were not examined since there was no appropriate validation assay available. Furthermore, the most commonly used reference miRNA, U6B, is not well characterized in rat. Using assays designed for human U6B quantification, we failed to detect it in our rat samples. 5S rRNA was instead used as reference RNA, since it is of similar size as miRNAs. Similar levels of 5S rRNA were observed in male and female liver (Figure 1). Based on qRT-PCR analysis, the expression of miR-451, miR-148a, miR-21, miR-205 and miR-193a were not different between the sexes and therefore contrasted the microarray results. High individual differences in miR-122a levels were observed in both males and females, and although the level of miR-122a seemed to be higher in females, this difference did not reach significance (2.3-fold, p = 0.09). However, miR-29b levels were significantly higher (2.1-fold, p = 0.03) in females as compared to males.

### Hormonal and nutritional regulation of hepatic miRNA

**Figure 2:**
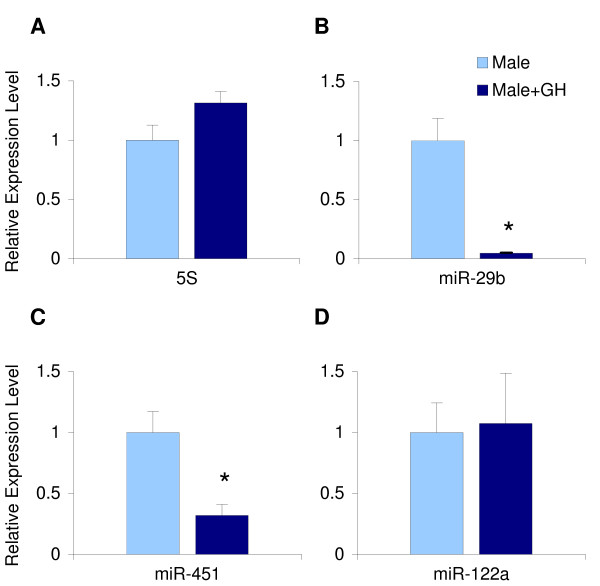
**Effects of GH treatment on hepatic miRNA levels**. Livers from untreated or GH-treated male rats were used to extract miRNA-enriched RNA. cDNA was synthesized from 100 ng of this miRNA-enriched RNA using miRNA specific primers (miR-29b, miR-451, miR-122a) or random hexamers (5S rRNA), and real-time PCR analysis was used to quantify RNA levels. The results were related to corresponding levels in untreated males and presented as ratios (mean ± S.E.). * indicate significant differences between untreated and GH-treated rats (p < 0.05) using student's t-test.

GH is one of the hormones that has been shown to regulate many male or female-predominant gene products in rat liver [11] and, due to its sex-different secretory pattern, to mediate sex-different activation of transcription factors of importance for this. To address the question whether the sex-different hepatic miRNAs could be regulated by GH, we determined their expression levels in male rats treated for one week with continuous infusion of GH through osmotic minipumps. This treatment has previously been shown to partly "feminize" hepatic gene expression [11]. miR-451 was significantly down-regulated (3.1-fold, p = 0.01) by GH (Figure 2c), whereas miR-122a expression was unaffected by this treatment (Figure 2d). High individual differences in miR-122a were again observed within the groups. Interestingly, miR-29b was drastically down-regulated by GH (21-fold, p = 0.008) (Figure 2b), GH-treatment did not affect the levels of 5S rRNA level (Figure 2a), suggesting a specific inhibitory effect of GH on miR-451 and miR-29b and putative roles for these miRNAs in the regulation of GH-dependent gene products.

**Figure 3:**
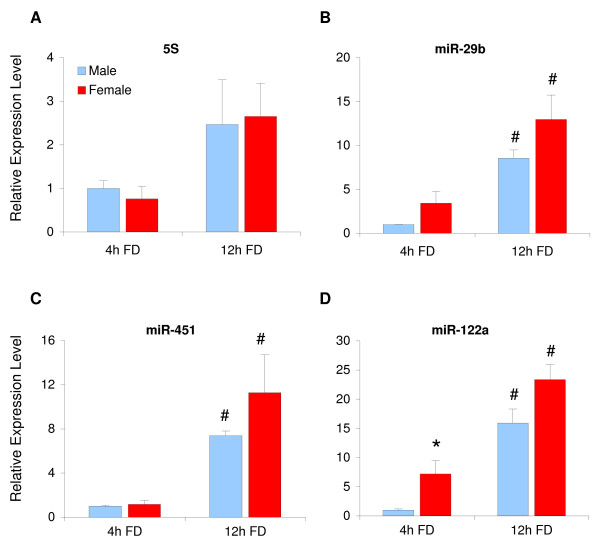
**Effects of mild starvation on hepatic miRNA levels**. Livers from mildly starved or postabsorptive male and female rats were used to extract miRNA-enriched RNA. cDNA was synthesized from 100 ng of this miRNA-enriched RNA using miRNA specific primers (miR-29b, miR-451, miR-122a) or random hexamers (5S rRNA), and real-time PCR analysis was used to quantify RNA levels. The results were related to corresponding levels in males at four hours of food deprivation and presented as ratios (mean ± S.E.). # indicate significant differences (p < 0.05) between mildly starved (12h FD) and postabsorptive (4h FD) rats, whereas * indicate significant sex-differences (p < 0.05), using student's t-test.

We have previously compared gene expression levels from rat livers during mild starvation (12 hours of food deprivation) and the postabsorptive state (4 hours of food deprivation) [11]. The notion that sex-dependent and GH-regulated transcripts were differentially expressed between these metabolic states, we next addressed the question whether miRNA levels would also be affected. As illustrated in figure 3, the level of miR-451 was significantly up-regulated in males (7.4-fold, p < 0.001) and females (9.7-fold, p = 0.03) after mild starvation, without any sex-differences (Figure 3c). Females had a significantly higher level (7.2-fold, p = 0.03) of miR-122a expression in the postabsorptive state, whereas mild starvation up-regulated miR-122a in both sexes and diminished this sex-difference (16-fold, p < 0.001 in males and 3.2-fold, p = 0.003 in females) (Figure 3d). Similar results were obtained for miR-29b, with increased levels in both males (8.5-fold, p < 0.001) and females (3-fold, p = 0.04) (Figure 3b). The level of 5S rRNA was also increased by mild starvation, but without statistical significance (Figure 3a). We conclude that mild starvation up-regulated the levels of miR-451, miR-122a and miR-29b significantly in both sexes, when compared to the postabsorptive state.

## Discussion

In this study, a miRNA screening was performed to identify sex-dependent miRNAs in rat liver. Among eight putative sex-different miRNAs, at least one female-predominant miRNA (miR-29b) could be confirmed using qRT-PCR. In contrast, the candidates for male-predominant miRNA could not be confirmed. In order not to miss any putative sex-dependent miRNA, a relatively high false discovery rate (q < 10%) and low cut-off (1.5) regarding differences between male and female expression levels were used. Moreover, the chemistry for detection by microarray and qRT-PCR are different. These might explain the inconsistent results between microarray and qRT-PCR, and stresses the importance of validating results obtained in this type of screening experiments.

miR-122a might be another female-predominant miRNA in rat liver, since significantly higher (7.4 times, p = 0.035) levels were observed in females than in males in the postabsorptive state. We observed smaller individual differences in miR-122a levels in these animals compared to animals with free access to food, which could be related to smaller individual differences in their metabolic states. This together with reports on multiple transcript variants of miR-122 [5] might explain the variation in miR-122a levels in livers from rats without food restriction. Sequence differences between these variants might be too small for qRT-PCR to discern.

The female-predominant expression of miR-29b and miR-122a observed in this study might be of importance for metabolic control, since these miRNAs were shown to be increased during mild starvation, a condition when the liver switches from anabolic to catabolic pathways. In an as yet unpublished study, we have observed that these mildly starved rats have lower hepatic glycogen content and higher rates of lipid turnover, as compared to the postabsorptive rats. Responses to mild starvation were also observed at the level of hepatic gene expression (unpublished results). However, since most transcript levels were independent of food deprivation, we do not believe that the data presented in figure 3 is a general effect of starvation on gene expression. Increased levels of e.g. miR-29b and miR-122a during starvation might reduce the translatability of key proteins for anabolic processes within the liver.

Among the miRNAs subjected to qRT-PCR, miR-29b was shown to be female-predominant and down-regulated in males given GH treatment. Continuous infusion of GH to male rats has previously been shown to mimic the female-specific endogenous secretion of GH and to partly "feminize" hepatic gene expression in rodents [14-16]. Obviously, miR-29b is not a transcript that is female-predominant due to the female-specific pattern of GH. Interestingly, there are also examples of male-predominant gene products that are induced in male livers upon GH treatment. Those transcripts include spot14, fatty acid synthase and stearoyl-CoA desaturase 1 [11], which are all involved in *de novo *lipid synthesis. This is in line with the observation that GH-treated male rats have higher hepatic triglyceride content [11].

To address the question of a putative biological significance of sex-different hepatic miRNAs, we searched for miRNA targets using the online databases PicTar [23] and TargetScan [24,25]. We found that the number of potential targets for different miRNAs differed greatly depending on the database (data not shown). miR-451 had only one predicted target in common between the databases, whereas miR-148a, miR-21 and miR-205 had 200 to 300 putative target genes. Comparing these lists of putative miRNA targets for miR29b or miR-122a towards transcript profiles generated in our lab comparing gene expression in male and female rat livers, no transcripts were in common. Based on a miRNA silencing study of miR-122 in mice, the inconsistency between *in silico *prediction analysis and *in vivo *data on miRNA targets is large [6]. This discrepancy between database-generated results and those obtained in biological experiments might be explained by the prediction algorithms used. The algorithms of the two selected databases are based on sequence similarities, so-called seed complementary. Hofacker argues that a thermodynamics-based prediction is a more reliable method than seed complementary to predict miRNA targets [26]. Nevertheless, *in vivo *studies should always be performed to validate any predicted functions and actions of miRNA.

Combining *in silico *and *in vivo *studies, He *et al *identified a potential miR-29 target, insulin-induced gene 1 (INSIG1, or growth response protein CL-6) [17]. INSIG1 is an endoreticulum-bound protein which inhibits the binding of SREBP to SREBP cleavage-activating protein (SCAP). In the absence of sterols, SCAP binds to SREBPs and mediates their transport from the endoreticulum (ER) to Golgi, where SREBPs are cleaved into their nuclear active form to induce lipid biosynthesis. INSIG1 binds to the sterol sensing domain of SCAP thus inhibiting its binding to SREBPs [27]. We have previously noticed that INSIG1 mRNA [12], INSIG1 protein, as well as SREBP-1 activation (unpublished data) is highly up-regulated by continuous GH-treatment in male rat livers. Since the main mechanism whereby miRNAs affect protein translatability is to inactivate/degrade the target mRNA, a lower level of miR-29b in response to GH might increase the amount of active/complete INSIG1 mRNA. Further experiments are required to address this.

## Conclusion

We have previously shown that hepatic transcript profiles are different between males and females; that some of these differences are under the regulation of GH; and that mild starvation diminishes some of the differences. In this study, we show that hepatic miRNAs are regulated in a similar manner.

## Methods

### Animals

Ten weeks old male and female Sprague-Dawley rats (B&K Universal AB) were maintained under standardized conditions. Five male rats were treated with bovine GH (bGH), a kind gift from Pharmacia and Upjohn AB, by continuous infusion from osmotic minipumps (model 2001; B&K Universal AB) at a dose of 5 μg/h. After one week of treatment, the rats were sacrificed and tissues removed and frozen in liquid nitrogen. When the effect of mild starvation was investigated, another group of age-matched male and female rats were used. Food was removed early in the morning (7 a.m.) or late in the evening (11 p.m.), so that the animals were without food 4 hours (postabsorptive) or 12 hours (mild starvation) before they were sacrificed (around noon). Animals were anesthetized, sacrificed, tissues removed and immediately frozen in liquid nitrogen and stored at -80°C until further analysis. All animal experiments were approved by the institutional animal care and use committee.

### miRNA isolation

miRNA-enriched RNA was extracted from liver using miRNeasy Mini kit (Qiagen), according to the manufacturer's protocol. In short, 25 mg of frozen liver was homogenized and extracted using phenol:chloroform. The resulting aqueous phase was mixed with 80% ethanol to extract RNA, followed by precipitation using 100% ethanol and high salt buffers. One of these buffers removes large RNAs (> 200 nucleotides). After a wash using 80% ethanol, miRNA-enriched RNAs were eluted using RNase-free water and quantified by a NanoDrop ND-1000 Spectrophotometer (NanoDrop Technologies Inc, USA, DE)

### Microarray

Four individual arrays were run for each sex. 3 μg of miRNA-enriched hepatic RNA derived from four males and four females were labeled and hybridized against each other as individual pairs onto the miRCURY™LNA (Locked Nucleic Acid) microRNA Arrays (208000V8.0, Exiqon, Danmark), according to the manufacturer's protocol. miRNA was considered to be expressed if it was detected in more than 75% of the individual array experiments with a reliable signal (signal to background ratio higher than 1.6). The data were normalized by the algorithm LOWESS using the program R and the significance of any sex-different expression was calculated using the program Significance Analysis of Microarray (SAM). miRNAs identified with a false discovery rate (q-value) lower than 10% and a male to female ratio higher than 1.5 were considered to be significantly sex-different. A p-value was calculated comparing the overall ratio to a value of 1.0, using student's t-test.

### Quantitative real-time PCR

Selected miRNAs (miR-122a, miR-29b and miR-451), and 5S rRNA as reference gene, were further examined using the miRCURY™LNA Real-time PCR kit (Exiqon, Danmark), according to the manufacturer's protocol. Briefly, 100 ng miRNA-enriched RNA was reverse-transcribed into cDNA using miRNA-specific primers for miRNA detection or random hexamer for the reference gene. The reverse transcription primer carries an additional sequence complementary to the 3'-end of the PCR primer. Together with a miRNA-specific LNA primer, the synthesized cDNA was amplified and detected by SYBR^®^Green. Detection and data analysis were performed on Applied Biosystems 7300 Real-Time PCR System and its associated software. Differential expression was determined by delta-delta Ct method that shows the fold changes of samples relative to a reference sample. Individual miRNA (from at least four livers from each animal group) were extracted and analyzed twice. The graphs show the average of at least eight data points for each group and the error bars represent their S.E.

## Authors' contributions

LC took part in the design of the study, carried out animal experiments, RNA isolation, cDNA synthesis and real-time PCR analyses, immunoblotting and drafting of the manuscript. CG took part in animal experiments, carried out RNA isolation, cDNA synthesis and microarray analyses. GN participated in design and coordination of the study, took part in animal experiments and helped to draft the manuscript. PTE participated in design and coordination of the study, took part in animal experiments and drafted the manuscript. All authors read and approved the final manuscript.
